# High temperature utilization of PAM and HPAM by microbial communities enriched from oilfield produced water and activated sludge

**DOI:** 10.1186/s13568-019-0766-9

**Published:** 2019-04-09

**Authors:** Carolina Berdugo-Clavijo, Arindom Sen, Mojtaba Seyyedi, Harvey Quintero, Bill O’Neil, Lisa M. Gieg

**Affiliations:** 1 Department of Biological Sciences, University of Calgary, 2500 University Drive NW, Calgary, AB T2N 1N4 Canada; 2 Department of Chemical and Petroleum Engineering, Schulich School of Engineering, University of Calgary, 2500 University Drive NW, Calgary, AB T2N 1N4 Canada; 3 Trican Well Service, 2900, 645-7th Avenue SW, Calgary, AB T2P 4G8 Canada

**Keywords:** Polyacrylamide, Hydraulic fracturing, Wastewater sludge, Biodegradation, PAM, HPAM

## Abstract

Non-hydrolyzed polyacrylamide (PAM) and partially hydrolyzed polyacrylamide (HPAM) are commonly used polymers in various industrial applications, including in oil and gas production operations. Understanding the microbial utilization of such polymers can contribute to improved recovery processes and help to develop technologies for polymer remediation. Microbial communities enriched from oilfield produced water (PW) and activated sludge from Alberta, Canada were assessed for their ability to utilize PAM and HPAM as nitrogen and carbon sources at 50 °C. Microbial growth was determined by measuring CO_2_ production, and viscosity changes and amide concentrations were used to determine microbial utilization of the polymers. The highest CO_2_ production was observed in incubations wherein HPAM was added as a nitrogen source for sludge-derived enrichments. Our results showed that partial deamination of PAM and HPAM occurred in both PW and sludge microbial cultures after 34 days of incubation. Whereas viscosity changes were not observed in cultures when HPAM or PAM was provided as the only carbon source, sludge enrichment cultures amended with HPAM and glucose showed significant decreases in viscosity. 16S rRNA gene sequencing analysis indicated that microbial members from the family *Xanthomonadaceae* were enriched in both PW and sludge cultures amended with HPAM or PAM as a nitrogen source, suggesting the importance of this microbial taxon in the bio-utilization of these polymers. Overall, our results demonstrate that PAM and HPAM can serve as nitrogen sources for microbial communities under the thermophilic conditions commonly found in environments such as oil and gas reservoirs.

**Electronic supplementary material:**

The online version of this article (10.1186/s13568-019-0766-9) contains supplementary material, which is available to authorized users.

## Introduction

Non-hydrolyzed polyacrylamide (PAM) and partially hydrolyzed polyacrylamide (HPAM) are frequently used in many industrial applications including waste water treatment processes (Guezennec et al. 2015), soil conditioning and erosion control (Levy and Warrington 2015), and oil and gas operations. In the latter sector, these polymers are used as thickening agents and friction reducers for enhanced oil recovery and hydraulic fracturing processes (Sorbie 2000; Montgomery 2013). However, when PAM or HPAM is injected into reservoirs to help transport proppants, these polymers can accumulate in the fractures and form “filter cakes” which cause formation damage that blocks the production of crude oil or gas (Prud’homme and Wang 1993). Chemical oxidizers, including sodium or ammonium persulfates, are commonly added to reservoirs to degrade these residual polymers (Montgomery 2013), but their use can lead to other issues including the corrosion of tubulars and safety/handling concerns. As an alternative strategy, enzyme-based breaker technologies have previously been shown to be effective for treating residual polymers such as guar gum (Brannon et al. 2003). Thus, the use of microbial activity or enzymes may also have utility in degrading residual PAM or HPAM added to oil and gas production operations.

The microbial utilization of PAM and HPAM has been studied since the early 1990s, mainly under mesophilic temperatures (e.g., between 20 and 39 °C) and with microorganisms enriched from soil, oilfields, or activated sludge. Kay-Shoemake et al. (1998a) observed that aerobic soil microorganisms utilized PAM as a nitrogen (N) source when incubated at 30 °C, but they did not grow when PAM was supplemented as a sole carbon (C) source. Extracellular amidases involved in the hydrolyzation or deamination of PAM were detected over time in these cultures (Kay-Shoemake et al. 1998b). Similarly, anaerobic microorganisms including sulfate-reducing bacteria (Grula et al. 1994; Hu et al. 2018) and methanogenic consortia (Haveroen et al. 2005; Hu et al. 2018) were shown to utilize HPAM as a N source when incubated between 22 and 37 °C. Bao et al. (2010) reported that microorganisms obtained from oilfield produced water were able to utilize HPAM as a source of N and/or C when incubated at 37 °C. However, the enzymes involved in the biodegradation of HPAM were not identified in this report. Changes in the molecular weight and viscosity of HPAM with microorganisms enriched from activated sludge have also been reported in recent studies (e.g., Dai et al. 2014; Yu et al. 2015; Sang et al. 2015; Yan et al. 2016; Zhao et al. 2016), although in some cases it is not clear whether these changes were due solely to biological degradation of the polymers because abiotic and HPAM-free controls were not described. Although the cleavage of the carbon–carbon bonds in PAM or HPAM has not been unequivocally demonstrated, the conversion of amide (NH_2_) groups into carboxylic acid (COOH) has been repeatedly shown using approaches such as FT-IR, LC/MS, and GC/MS analyses (Ma et al. 2008; Liu et al. 2012; Dai et al. 2014; Yu et al. 2015; Sang et al. 2015).

The microbial utilization of PAM has been shown to be affected by various factors including the polymer concentration, pH, and the presence of other C sources. Wen et al. (2010) observed the highest amide removal efficiency (70%) from PAM when 50 to 200 mg/L of PAM were amended to microorganisms enriched from soil, but showed that higher concentrations of PAM (> 200 mg/L) decreased the hydrolyzation efficiency in the cultures. Furthermore, in microbial communities obtained from waste water sludge, the addition of glucose was reported to increase PAM utilization (Wen et al. 2010; Dai et al. 2014; Yu et al. 2015; Yan et al. 2016). Yan et al. (2016) reported that the highest hydrolyzation activity (up to 50%) in HPAM was reached when 100 mg/L of glucose were added to a bioreactor activated sludge culture at an initial pH of 8.0. Similarly, Yu et al. (2015) observed that the addition of glucose increased the microbial hydrolysis of PAM up to 20%. Moreover, Dai et al. (2015) observed that in anaerobic active sludge bioreactors with an initial pH of 9.0, the addition of either starch or bovine serum albumin increased PAM hydrolysis up to 39%. Other factors that may influence microbial utilization of PAM or HPAM could be temperature and degree of polymer hydrolyzation. However, these factors have not yet been well explored.

Produced waters from hydraulic fracturing operations and waste water sludges are commonly exposed to polymers such as PAM or HPAM at relatively high temperatures (> 50 °C). Thus, we hypothesized that these environments harbour microbial communities able to biodegrade these polymers under thermophilic conditions. In this study, we investigated the potential for the microbial utilization of PAM and HPAM as C and/or N sources at 50 °C using microbial communities enriched from oilfield produced water and activated sludge as a screening approach to identify possible activities or enzymes that can be used to biodegrade these polymers that are often used in hydraulic fracturing and enhanced oil recovery operations in situ.

## Materials and methods

### Establishment of microbial enrichments

**Fig. 1:**
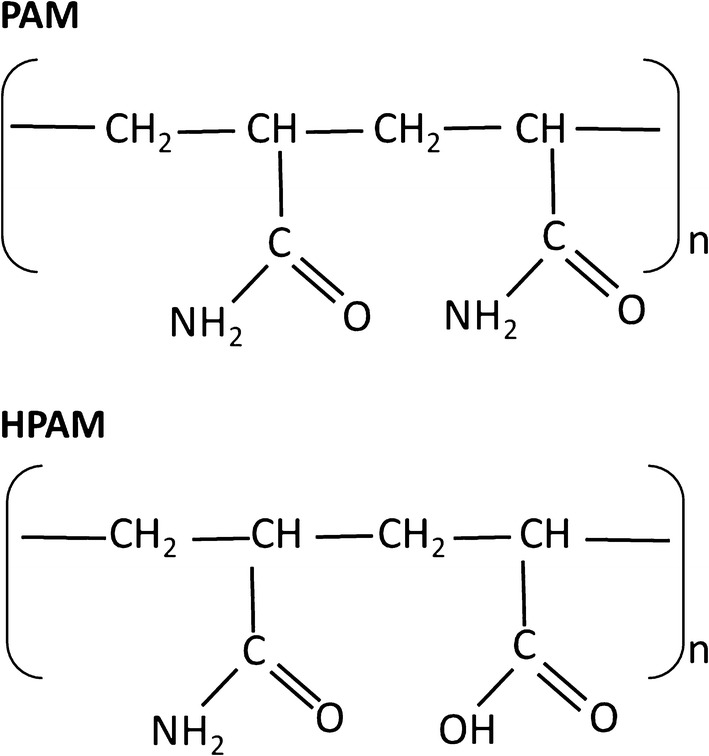
Chemical structures of PAM and HPAM, representing the polymers used in this study

Produced water (PW) from a hydraulic fracturing well in Alberta, Canada and activated sludge from a wastewater treatment facility in Alberta, Canada were used to enrich for microbial cultures able to utilize PAM and 25–30% HPAM obtained from an industrial source (Fig. 1). The PW sample contained between 0.1 and 1% of BTEX compounds (benzene, toluene, ethylbenzene, and xylenes) and petroleum components (traces) and had a salinity of 0.9%. For the preparation of the initial microbial enrichment culture, PW (50 mL) was combined with 150 mL of nutrient medium containing the following ingredients (per liter of water): 0.5 g KH_2_PO_4_, 0.5 g K_2_HPO_4_, 0.2 g MgSO_4_, 5 g NaCl, 5 g yeast extract, 10 g tryptone, and 50 mg of PAM. The initial enrichment culture was incubated on a shaker (120 rotations per minute, rpm) at 50 °C for up to 7 days. The microbial culture (5 mL) was then transferred to 50 mL of minimal medium containing the following (per liter of water): 0.5 g KH_2_PO_4_, 0.5 g K_2_HPO_4_, 0.2 g MgSO_4_, 5 g NaCl, 5 g yeast extract (only added for the first transfer), 14.7 mg CaCl_2_, and 1 mL trace metals (Kay-Shoemake et al. 1998b), 5 mL vitamin solution (Atlas 2010), and 250 mg/L PAM as a source of carbon (C) and nitrogen (N). For the initial preparation of the activated sludge enrichments, 25 mL of sludge were combined with 25 mL of minimal medium (described above) and spiked with 50 mg/L PAM or HPAM. All enrichments were incubated at 50 °C on a shaker (120 rpm) for up to 22 days. Both PW and sludge enrichments were transferred at least three times to dilute out other sources of C originally present in the samples by taking 5 mL from each enrichment and mixing it with 50 mL of fresh minimal medium containing 250 mg/L of PAM or HPAM.

### PAM and HPAM as carbon and/or nitrogen sources

The established microbial enrichments of PW and activated sludge (described above) were used as sources of inocula to test whether microorganisms present in these cultures were able to utilize PAM and HPAM as C and/or N sources. Incubations were prepared in 160 mL serum bottles containing 50 mL minimal medium. A set of incubations was amended with 500 mg/L of either PAM or HPAM as the C source, and with 100 mg/L of NaNO_3_ as a N source. A second set of incubations was prepared with 500 mg/L of glucose as the C source, and 250 mg/L of either PAM or HPAM as the only N source. For the preparation of the microbial inocula, cell pellets from a total of 100 mL of PW enrichment cultures were harvested by centrifugation at 10,000×*g* for 15 min and re-suspended in 50 mL of fresh minimal medium. For the sludge enrichments, a total of 150 mL of sludge microbial culture was centrifuged and resuspended in the same manner as for the PW inoculum. Each incubation was inoculated with 5 mL of harvested cells (cell numbers not determined) from either PW or activated sludge cultures. Parallel controls were prepared in an identical manner, either without the addition of any added C source (substrate-free controls), or without microbial inoculum (abiotic controls). Triplicates were prepared for each treatment and control incubations. All incubations were aerobic, under a headspace of atmospheric air, sealed with Teflon stoppers and incubated at 50 °C, with shaking at 120 rpm for up to 34 days.

### Physical and chemical analyses

Microbial growth in PW and activated sludge incubations was monitored by measuring headspace CO_2_ production over time. Optical density measurements frequently used to monitor microbial growth were not possible for these cultures because the polymers themselves created turbid conditions in the incubations. CO_2_ was measured using a gas chromatograph (GC) equipped with a thermal conductivity detector (Agilent 7890A) and a HP-PLOT/Q capillary column (30 m). The oven temperature was held at 80 °C for 5 min, the detector was at 200 °C, and the inlet was held at 250 °C. The final concentration of CO_2_ gas was calculated based on a calibration curve prepared from CO_2_ standards analyzed under the same conditions as the samples. Theoretical CO_2_ production was calculated for the amount of glucose added in the cultures, assuming complete mineralization to CO_2_ (Table 1). Oxygen concentrations were also monitored in the microbial cultures to ensure that aerobic conditions were maintained during the incubation period (using the same GC method). Statistical analysis was conducted for CO_2_ production results using a one-way ANOVA (McDonald 2014) and the Tukey–Kramer test to identify significant differences (p < 0.05) between enrichments and polymer free controls after 34 days of incubation, where minimum significant difference is calculated for each pair of means.

**Table 1:** Theoretical stoichiometry of glucose oxidation in the HPAM and PAM microbial cultures

$${\text{C}}_{ 6} {\text{H}}_{ 1 2} {\text{O}}_{ 6} + 5.685{\text{O}}_{ 2} + 0.06{\text{NH}}_{3} \to 0.3{\text{CH}}_{1.8} {\text{O}}_{0.5} {\text{N}}_{0.2} + 5.7{\text{CO}}_{2} + 5.84{\text{H}}_{2} {\text{O}}^{\text{a}}$$		
Culture volume (mL)	Glucose added (μmol)	Theoretical CO_2_ expected (μmol)
50	139	792

^a^Biomass empirical formula was taken from Shuler and Kargi (2008). Stoichiometric calculation for biomass utilization was based on the study by Edwards and Grbić-Galić (1994)

Polymer utilization in the microbial enrichments amended with PAM or HPAM was determined on the same set of microbial enrichments using a starch-cadmium iodide assay (Lu and Wu 2001). This colorimetric method determines the presence of NH_2_ groups in acrylamide-based polymers. Thus, the assay can detect the removal of NH_2_ from PAM or HPAM, but it does not measure C skeleton breakage in these polymers. To conduct the assay, samples (1 mL) were collected over time and centrifuged at 10,000×*g* for 7 min. A volume of 200 µL of supernatant was then diluted with 1800 µL of distilled water, and other assay reagents were subsequently added as described by Lu and Wu (2001). HPAM and PAM standards of known concentrations were prepared in parallel to make calibration curves. Absorbance readings of the starch-triiodide blue complex were measured on a spectrophotometer at 540 nm. The percentage of NH_2_ removal was calculated by subtracting the measured concentration of the cultures at the end of the incubation from the concentration of the abiotic controls divided by the control concentration. Statistical differences in NH_2_ removal were identified between microbial enrichments (PW vs. sludge) and polymers (PAM vs. HPAM) using a paired T-test, wherein a *p*-value < 0.05 indicates a significant difference.

A new set of incubations was prepared to assess changes of viscosity in the PW and sludge microbial cultures using the same minimal medium described above and adding 500 mg/L of either PAM or HPAM as N source, and 500 mg/L of glucose as a C source. A higher concentration of polymer was used for this analysis to simplify viscosity measurements. Here, 1 mL of liquid culture was collected and analyzed using a bench-top viscometer (Brookfield LDV-II+P CP). Viscosity was measured (40 °C, 90 rpm) using a CP42 cone spindle. Statistical differences in viscosity values were determined by comparing values before and after incubation using a paired T-test, where a *p*-value < 0.05 indicates a significant difference. In addition, several attempts to measure the size distribution of the HPAM and PAM polymers were done using a Malvern Zetasizer Nano ZS as described by Kakadjian et al. (2013). However, optical interference did not allow for reliable results, despite efforts to improve the measurements (e.g., via centrifugation).

Organic acids such as propionate and butyrate (previously shown to be metabolites of PAM biodegradation, Dai et al. 2015) were assayed in the microbial cultures by high performance liquid chromatography (HPLC) (Waters 525 Model-Milford, MA). The HPLC was equipped with a UV detector (Waters 2487) set at 210 nm, and an organic acid column (250 × 4.6 mm, Alltech Prevail) eluted with KH_2_PO4 (25 mM, pH 2.5) with the mobile phase at 1 mL/min. Liquid samples (1 mL) were centrifuged at 10,000×*g* for 7 min. The supernatant was then passed through a 4 µm filter unit to filter out polymers in the samples. Finally, 300 µL of sample were mixed with 20 µL of H_3_PO_4_ and injected (50 µL) into the HPLC. To confirm the identity of the organic acids and to search for other possible metabolites formed, cultures were also extracted with ethylacetate as described by Berdugo-Clavijo et al. (2012), concentrated to 500 µL, and silylated with 50 µL *N*, *O*-bis-(trimethylsilyl) trifluoroacetamide (BSTFA, Thermo Scientific, Waltham, MA) for 20 min at 60 °C. Samples (1 µL) were injected into a gas chromatograph/mass spectrometer (GC/MS; model 7890A GC and model 5974 MS, Agilent, Santa Clara, CA). The inlet temperature was held at 270 °C. The oven was initially held for 5 min at 45 °C, then the temperature was increased (5 °C/min) up to 300 °C and held for 5 min. Authentic standards of various organic acids were prepared to compare retention times and MS profiles with any putative metabolites formed in the cultures.

### Microbial community structure of PW sample and PAM enrichments

**Fig. 2:**
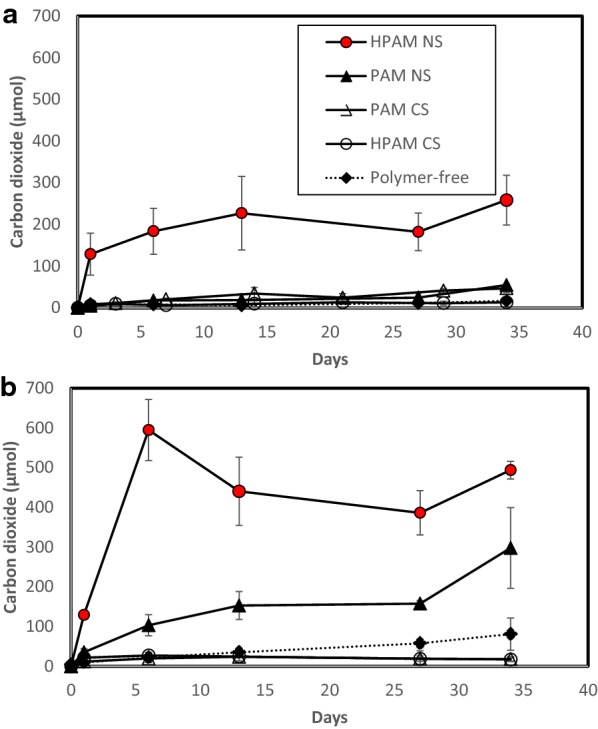
Carbon dioxide levels over time in microbial cultures obtained from **a** PW and **b** sludge enrichments after 34 days of incubation at 50 °C. HPAM/PAM NS: polymers were added as N source (glucose was the C source). HPAM/PAM CS: polymers were added as a sole C source (sodium nitrate was the N source). Error bars indicate standard deviations of triplicate samples

The microbial community compositions in the original PW and activated sludge samples, and their corresponding enrichment cultures amended with HPAM or PAM (same cultures used for the data shown in Fig. 2) were characterized based on 16S rRNA gene analysis. Genomic DNA was extracted from cell pellets collected from 3 to 15 mL of original samples or enrichment cultures using a commercial DNA extraction kit (Fast-DNA Spin Kit for Soil, MP Biomedicals). Isolated DNA was prepared for Illumina Miseq sequencing analysis by amplifying the 16S rRNA gene (V6–V8 region) with a 2-step PCR protocol using KAPA HiFi HotStart PCR kit (Biosystems). The first PCR reaction (25 µL) included the universal primers 926Fi5 (TCGTCGGCAGCGTCAGATGTGTATAAGAGACAGAAACTYAAAKGAATTGRCGG) and 1392Ri5 (GTCTCGTGGGCTCGGAGATGTGTATAAGAGACAGACGGGCGGTGTGTRC), and was amplified using a protocol suggested by KAPA manufacturer including a denaturation step at 98 °C for 20 s, and annealing at 65 °C for 15 s with 25 cycles. For the second PCR reaction (50 µL), Nextera XT indices (P5-S50X-OHAF and P7-N7XX-OHAF; Illumina) were attached to amplicon ends in preparation for Illumina MiSeq sequencing using the same PCR program described by Toth and Gieg (2018). PCR products of expected length were confirmed on a 1% agarose gel and purified after each PCR reaction using the Agencourt AMPure XP (Beckman Coulter) purification kit. Samples were normalized to a final concentration of 4 nM before running through the 300PE (pair-ended) MiSeq sequencing protocol at the University of Calgary, Canada. Overhang adapters and primers were removed from obtained raw sequences using the Cutadapt software (Martin 2011). Quality control analyses and read assembly were conducted in QIIME version 1.9.0 (Quantitative Insights Into Microbial Ecology, Caporaso et al. 2010). Raw reads were merged with a 50 bp (base pair) overlap, allowing less than 10% mismatches and allowing an average Quality Score below 20. Reads were clustered into operational taxonomic units (OTUs) with a 97% cut-off. Obtained OTUs were classified against the SSU SILVA database (SILVA 132) to assign specific taxonomy. Raw read sequences from PW and sludge enrichments were deposited in the GenBank archive at the National Center for Biotechnological Information (NCBI) under the following Accession Numbers (SUB4856039): MK246951, MK246952, MK246953, MK246954, MK246955, MK246956, MK246957, MK246958, MK246959, MK246960, MK246961, MK246962, MK246963, MK246964, MK246965, MK246966, MK246967, MK246968, MK246969, MK246970, MK246971. Sample names corresponding to each accession number are shown in Additional file 1: Table S4.

## Results

### Microbial growth in enrichment cultures

Microbial enrichment cultures from PW and activated sludge were established in the presence of PAM and HPAM (or in the absence of the polymers) and were repeatedly transferred until CO_2_ production was no longer detected in the substrate-free controls, indicating that background C and N sources (e.g., from the original field samples) were not present (data not shown). These microbial enrichments were tested for their ability to utilize HPAM and PAM as C and N sources at 50 °C. When HPAM or PAM was added to the PW and sludge microbial cultures as the only C source, microbial growth was very limited as indicated by the low CO_2_ production in these incubations (Fig. 2). For instance, in the PW-derived culture, incubations with PAM as the sole C source produced up to 47 ± 10 µmol of CO_2_ after 34 days of incubation, and 13.2 ± 5 µmol of CO_2_ were produced in cultures amended with HPAM as a C source (Fig. 2a). CO_2_ production in the cultures amended with PAM (as a C source) was significantly different than the controls without PAM, but there was not a significant difference between the culture with HPAM (added as a C source) and the HPAM-free control (Additional file 1: Table S1). For the sludge enrichments, 24 ± 10 µmol of CO_2_ were produced in incubations amended with PAM and up to 27 ± 2.3 µmol of CO_2_ were produced in the culture amended with HPAM (Fig. 2b). For the latter cultures, no significant difference was observed between the cultures and the substrate-free controls (Additional file 1: Table S1). In contrast, when HPAM was amended as the only N source (with glucose serving as the C source), microbial growth was highly stimulated in both PW- and sludge-derived enrichment cultures (Fig. 2). For example, when incubated with HPAM, the PW culture produced up to 258 ± 60 µmol of CO_2_ (Fig. 2a, Day 34) while the sludge culture produced up to 594 ± 77 µmol of CO_2_ (Fig. 2b, Day 6). Stoichiometrically, and accounting for 5% of carbon typically used for biomass (Edwards and Grbić-Galić 1994), these amounts of CO_2_ indicate that up to 75% of the added glucose was consumed in the sludge cultures and up to 33% of the glucose was used in the PW cultures (Table 1). Addition of PAM as a N source in the PW-derived enrichment did not enhance CO_2_ production relative to the polymer-free controls (Fig. 2a), while up to 297 ± 100 µmol of CO_2_ were measured in sludge cultures with PAM after 34 days of incubation (Fig. 2b). Statistical analysis showed that all cultures amended with either PAM or HPAM as a N source had a significant difference in CO_2_ production compared to the polymer-free controls (Additional file 1: Table S1). Moreover, CO_2_ accumulation in the sludge enrichments amended with either HPAM or PAM was higher than CO_2_ production in the PW enrichments (Fig. 2) suggesting that the community enriched from the sludge sample had greater capability for polymer utilization.

### Microbial utilization of PAM and HPAM

**Fig. 3:**
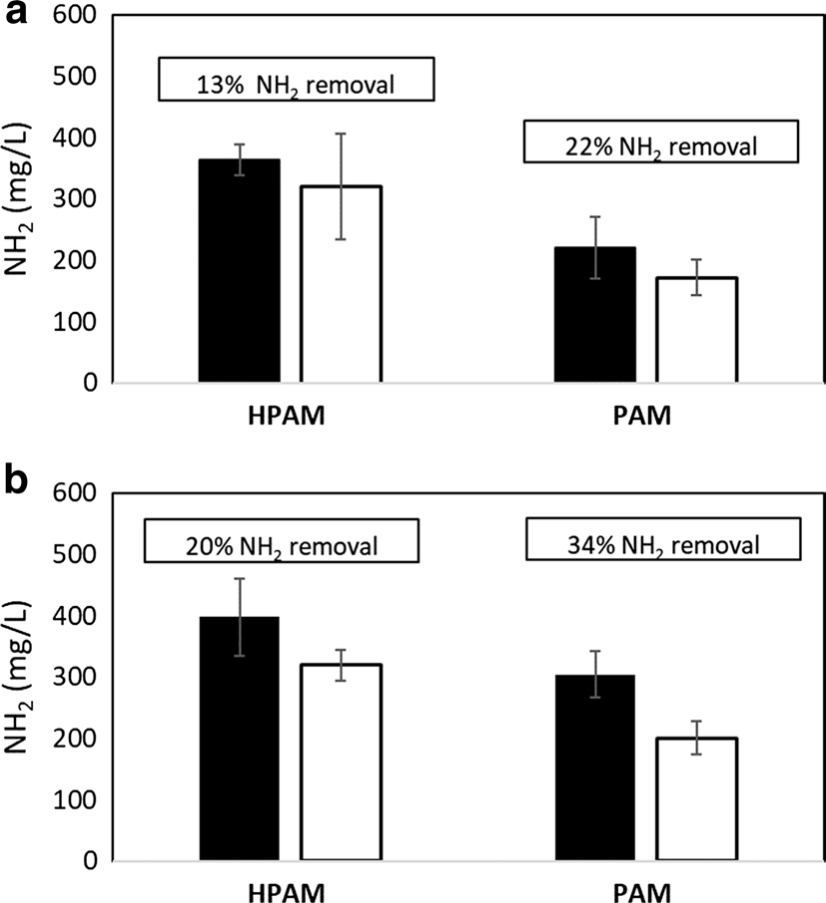
NH_2_ concentrations in **a** PW and **b** sludge microbial cultures amended with HPAM or PAM as a N source after 34 days of incubation. Black bars: sterile controls and white bars: actual cultures. Percentage of NH_2_ removal is also indicated for each enrichment

The microbial utilization of NH_2_ in the polymers was determined in the microbial cultures amended with PAM or HPAM as a N source (from same cultures used to generate the data shown in Fig. 2). The percentage of NH_2_ removed in the PW cultures amended with PAM or HPAM was 22 and 13%, respectively (Fig. 3a). In the sludge cultures, up to 20% NH_2_ removal was observed in the cultures amended with HPAM, while 34% NH_2_ removal was observed for the cultures containing PAM (Fig. 3b). These results indicate that both HPAM and PAM were deaminated by the microbial enrichment cultures. In the PW microbial cultures, significant differences between controls and cultures amended with PAM were observed (*p*-value: 0.003), while no significant differences (*p*-value: 0.4) were observed between controls and cultures amended with HPAM. A higher percentage of removal was observed in the cultures with PAM, possibly because there are more NH_2_ groups in the PAM molecule compared to HPAM (Fig. 1). No significant differences between controls and cultures were observed in the sludge enrichments (HPAM, *p*-value: 0.6 and PAM, *p*-value: 0.4), despite apparent removals.

**Fig. 4:**
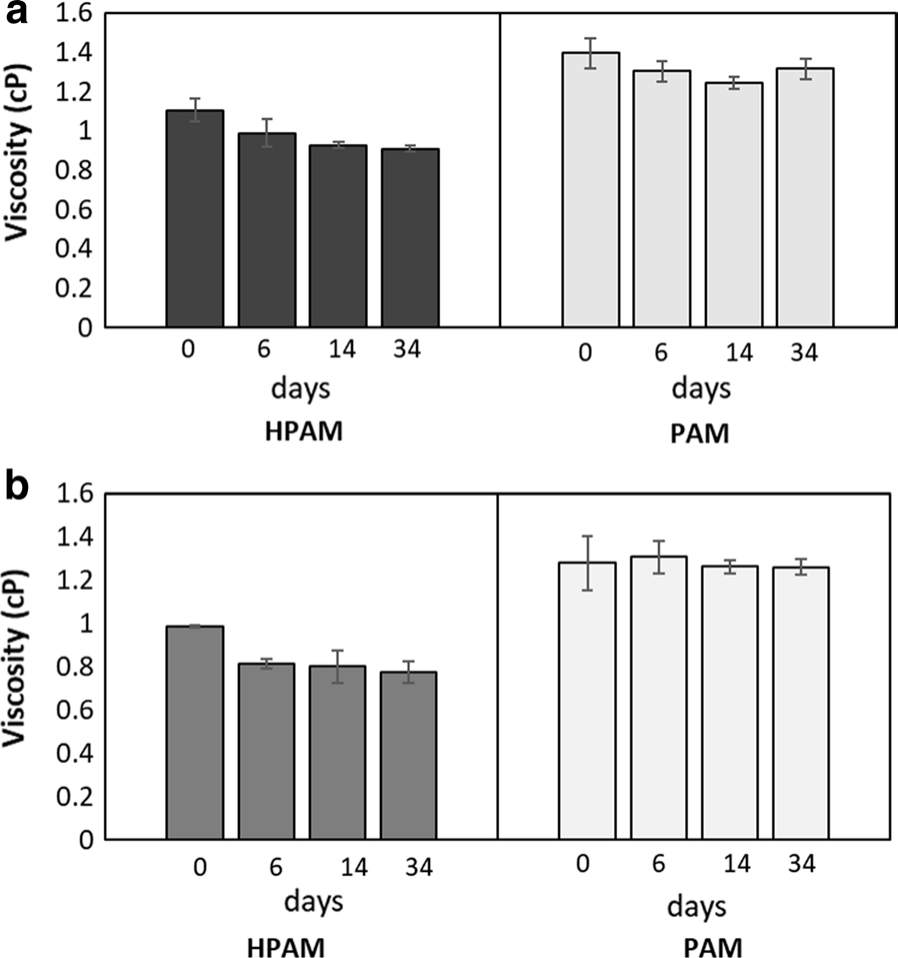
Viscosity measured in microbial enrichments derived from **a** PW and **b** sludge amended with PAM or HPAM as a N source at 50 °C. Viscosity was measured at 40 °C and 90 rpm. Error bars indicate standard deviations of triplicate samples

Viscosity was measured in all microbial enrichments amended with HPAM or PAM as a N or C source. Viscosity changes were not observed when HPAM or PAM was supplied as a sole C source in either the PW or sludge incubations (data not shown). Similarly, insignificant reductions in viscosity were observed when PAM served as a N source in PW or sludge enrichments (Fig. 4a, b, *p*-values: 0.07 and 0.8, respectively). In contrast, higher viscosity changes were observed in the microbial enrichments amended with HPAM as a N source. Viscosity decreased by up to 18% (*p*-value: 0.014) in the PW culture (Fig. 4a), and up to 21% (*p*-value: 0.008) in the sludge enrichment after 34 days (Fig. 4b). To explore whether the viscosity reduction in the sludge culture was a result of the biodegradation of HPAM (e.g., C–C cleavage), organic acids such as propionate and butyrate reported as possible biodegradation products (Dai et al. 2014), were sought in these cultures. Initial analysis by HPLC showed a peak with the same retention time as butyrate and propionate standards. However, further GC/MS analysis revealed that neither butyrate nor propionate were present in the samples, as mass spectra did not match with those of authentic standards. Further, no other putative metabolites potentially formed from HPAM biodegradation could be detected by GC/MS relative to controls, thus C utilization from the polymer could not be concluded.

### Microbial community structure in PAM and HPAM enrichments

**Fig. 5:**
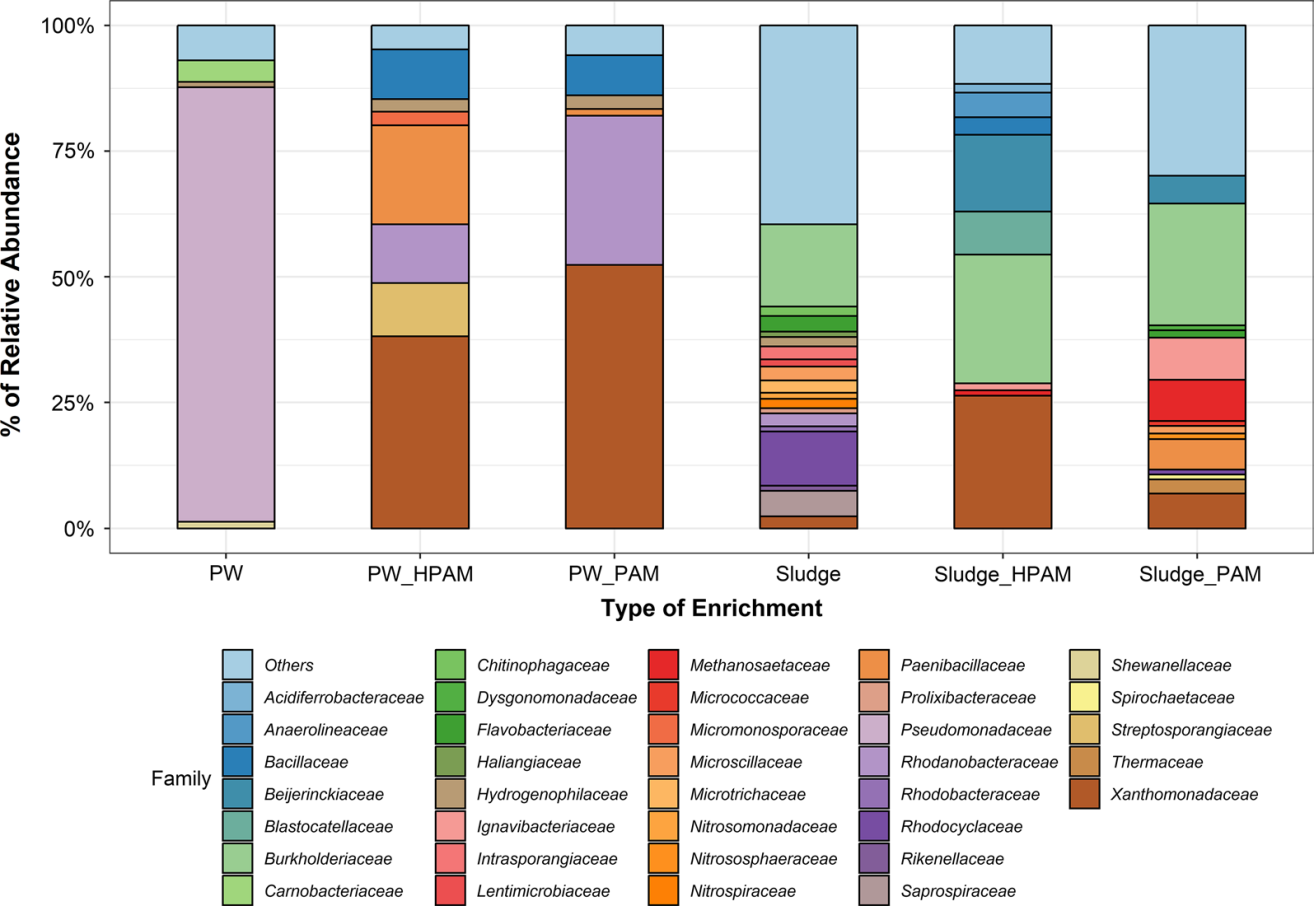
Microbial community compositions of the original oilfield PW sample (PW), PW enrichments (PW_HPAM, PW_PAM), wastewater sludge sample (Sludge), and sludge enrichments (Sludge_HPAM and Sludge_PAM), based on the relative abundances of taxa identified at the family level (only OTUs > 1% of the total reads are shown). Total number of reads in PW: 128,354, PW_HPAM: 11,1687, PW_PAM: 66,474, Sludge: 52,795, Sludge_HPAM: 24,656, and Sludge_PAM: 3836. Taxa with relative abundance < 1% or that could not be identified at the family level are grouped together as ‘Others’

The microbial community compositions of the original PW and sludge samples were compared to those of the microbial enrichments amended with HPAM or PAM as N sources (e.g., performed on the same cultures as those used for the other measurements, shown in Fig. 2). The PW sample was dominated by members of the *Pseudomonadaceae* family (86% relative abundance) (Fig. 5), comprised mainly of *Pseudomonas* species (Additional file 1: Table S2). Other microbial members present in lower proportions in this sample included *Desemzia, Shewanella, Tepidiphilus,* and *Acinetobacter* species which belong to the families *Carnobacteriaceae*, *Shewanellaceae*, *Hydrogenophilaceae*, and *Moraxellaceae*, respectively. After PW was supplemented with HPAM (PW_HPAM) or PAM (PW_PAM) as a N source, the community composition drastically shifted (Fig. 5). Members of the *Xanthomonadaceae* family became most abundant in both PAM- or HPAM-amended cultures (Fig. 5), mainly dominated by *Pseudoxanthomonas* phylotypes (Additional file 1: Table S2). Members of the *Xanthomonadaceae* family was found at a slightly higher abundance (52%) in the culture supplemented with PAM. Other microbial members that increased in abundance in PAM or HPAM PW cultures included phylotypes within the *Rhodanobacteraceae*, *Bacillaceae*, and *Hydrogenophilaceae* families (Fig. 5). Microbial members that were only enriched in the PW culture amended with HPAM included *Paenibacillaceae*, *Streptosporangiaceae*, and *Micromonosporaceae* (Fig. 5).

The microbial community composition of the wastewater sludge sample was not dominated by a specific microbial member; instead microbial members (< 16% relative abundance) belonging to various families were present including those from *Burkholderiaceae*, *Rhodocyclaceae*, *Saprospiraceae*, *Flavobacteriaceae*, *Intrasporangiaceae*, and *Microtrichaceae* (Fig. 5). In addition, most of the microbial members within these families were not identified at the genus level (Additional file 1: Table S3). Interestingly, after the sludge sample was amended with HPAM and PAM, the relative abundance of microbial members belonging to the family *Xanthomonadaceae* increased in both enrichments. For instance, in the sludge enrichment, the relative abundance of *Xanthomonadaceae* members increased from 2.4 to 26% relative abundance in the culture amended with HPAM (Fig. 5). In the culture amended with PAM, members of the *Burkholderiaceae* family represented up to 24% of relative abundance, but in this culture *Xanthomonadaceae* members were also increased in abundance (up to 7%) (Fig. 5). Other members that were enriched in the sludge culture amended with HPAM belonged to the families *Beijerinckiaceae*, *Blastocatellaceae*, *Anaerolineaceae*, and *Bacillaceae* (Fig. 5). Within these families, microorganisms identified at the genus level included *Chelatococcus, Bellilinea,* and *Anoxybacillus* species (Additional file 1: Table S3).

## Discussion

Previous studies have shown the ability of microorganisms to utilize PAM and HPAM under mesophilic conditions (e.g., between 20 and 37 °C), but this is the first report to show that microbial PAM or HPAM utilization is also possible under thermophilic conditions (50 °C). Here, microbial communities enriched from oilfield PW and wastewater sludge were shown to readily utilize PAM and HPAM as N sources at 50 °C, which was confirmed by the CO_2_ accumulation in the cultures (Fig. 2) and the partial removal of NH_2_ groups from the polymers (Fig. 3). In contrast, microbial communities enriched from PW or sludge were not able to grow when incubated with PAM or HPAM as the sole C source. Microbial utilization of PAM or HPAM as the only C source was reported under mesophilic temperatures (Wen et al. 2010; Bao et al. 2010; Dai et al. 2014; Yu et al. 2015), but unequivocal evidence showing the cleavage of the carbon–carbon bonds in PAM or HPAM has been more difficult to demonstrate. Dai et al. (2014) reported that volatile fatty acids (VFAs), including acetic, valeric, butyric, and propionic acids, accumulated over time in anaerobic cultures utilizing PAM as the only C source in a dewatered sludge system. Similarly, Hu et al. (2018) reported the formation of formate and acetate in anaerobic microbial cultures enriched from oilfield water when HPAM was the only C source.

We observed that in the sludge enrichments amended with HPAM and glucose, HPAM viscosity significantly decreased over time (Fig. 4). However, we could not unequivocally confirm whether these viscosity changes resulted from the cleavage of the C–C bonds in HPAM due to two main reasons. First, significant changes in viscosity of HPAM-amended cultures were only observed in cultures that had glucose present and not in cultures when HPAM was added as a sole C source. Second, we did not detect any putative metabolites potentially resulting from HPAM biodegradation (e.g., propionate, butyrate, or other potential products) by either the HPLC or GC/MS methods used. As these microbial cultures showed the highest accumulation of CO_2_ (Fig. 2b), we speculate that the significant decrease in viscosity may have been an effect of CO_2_ production. Microbial products including CO_2_ are known to decrease viscosity in crude oils (Bryant and Burchfield 1989), and more recently it was shown that supercritical CO_2_ can also reduce viscosity in polymer solutions (Lee et al. 2000). Bao et al. (2010) proposed that HPAM biodegradation can occur via oxidation reactions wherein –OH groups are added to the alpha carbon of the polymer, resulting in the formation of =O group and the cleavage of carbon bonds; but the presence and activity of such monooxygenases were not demonstrated in the report. Dai et al. (2015) indicated that enzymes presumably involved in PAM metabolism were present in anaerobic cultures able to utilize PAM; however, many of these enzymes are known to be associated with glucose metabolism, and glucose was also added to the incubations in that study. More recently, Song et al. (2018) detected laccase (an oxidase) and dehydrogenase activities in aerobic and anaerobic bioreactor systems able to utilize HPAM in the absence of glucose. Overall, enzymes involved in the hydrolyzation of HPAM or PAM have been widely detected in microbial cultures able to utilize the polymer as a N source (Kay-Shoemake et al. 1998b; Yu et al. 2015). However, enzymes able to catalyze the cleavage of the carbon bonds in PAM or HPAM have only been recently reported and they should be carefully identified, especially when additional C sources are added at some point in the microbial systems used as inocula.

Interestingly, microbial communities enriched from the wastewater sludge produced higher amounts of CO_2_ than microbial enrichments derived from PW (Fig. 2), showing that the sludge-derived microbial communities have a greater ability to utilize PAM or HPAM as a N source. Although a slightly higher percentage of NH_2_ removal was obtained for cultures amended with PAM (likely because higher NH_2_ groups are present in this polymer), PW or sludge microbial cultures had a higher microbial activity (CO_2_ accumulation) when amended with HPAM as a N source relative to cultures amended with PAM (Fig. 2). Our results showed that the microbial cultures enriched from PW and sludge had a higher tolerance and metabolic preference for HPAM. A similar result was also reported by Grula et al. (1994) who observed a slightly higher microbial activity (OD_540_ = 0.68) in sulfate-reducing cultures when they were amended with 25–35% hydrolyzed PAM compared to the activity observed with 1–4% hydrolyzed PAM (OD_540_ = 0.48). Collectively, these results suggest that microbial activity (e.g., growth, C metabolism, N utilization) is higher when exposed to PAM molecules containing a higher percentage of hydrolyzation.

Although the microbial community compositions of the PW and sludge samples were initially very different, both showed a substantial enrichment in microorganisms affiliating with the family *Xanthomonadaceae* (within the *Proteobacteria*) after the samples were amended with HPAM as a N source. Specific members of the *Xanthomonadaceae* have not been previously observed as HPAM or PAM utilizers, but *Proteobacteria* including *Acinetobacter*, *Pseudomonas*, *Azomonas, Desulfovibrio,* and *Enterobacter* species have been detected in microbial communities capable of utilizing PAM or HPAM (Grula et al. 1994; Nakamiya and Kinoshita 1995; Matsuoka et al. 2002; Li et al. 2015; Yu et al. 2015). In addition, members of the *Pseudoxanthomonas* sp. were highly increased in abundance in the HPAM-amended sludge enrichment where significant viscosity changes were observed. *Pseudoxanthomonas* species were previously shown to produce biosurfactants with high emulsifying activities (Nayak et al. 2009). Thus, it may be possible that the significant viscosity reduction observed in the sludge enrichment amended with HPAM was due to biosurfactant production in the cultures (in addition to the possible CO_2_ effects described above), however additional studies are needed to investigate this. Further, members of the *Xanthomonadaceae* family have been shown to be involved in acrylamide biodegradation. Nawaz et al. (1993) isolated an acrylamide-degrading microorganism identified as *Xanthomonas maltophilia*, and amidase genes related to acrylamide utilization were identified in *Xanthomonas campestris* (Chin et al. 2007). Our work thus adds to the growing body of knowledge showing that members of *Xanthomonadaceae* play a key role in acrylamide-based polymer utilization.

Overall, we observed that under thermophilic conditions (50 °C), microorganisms enriched from oilfield PW and wastewater sludge have the ability to utilize PAM and HPAM as N sources. Although our carefully controlled experiments showed that these microbial communities reduced HPAM viscosity when glucose was present, we were not able to unequivocally conclude that carbon–carbon cleavage of HPAM occurred in these cultures as no putative metabolites could be detected relative to controls and size fraction measurements were inconclusive. As postulated, though, CO_2_ and/or biosurfactant production may play a role in viscosity reduction but further study is required. However, our work did show that PAM and HPAM can readily stimulate microbial activity in environments at relatively high temperatures (50 °C) by providing a N source for microbial communities when additional C sources are available (such as hydrocarbon components or carbohydrates), potentially resulting in viscosity reduction. Such microbial activity resulting from the utilization of the NH_2_ portions of PAM and HPAM could have either beneficial or negative effects in oil production operations, thus these polymers should be considered as potential nutrients sources for microorganisms living in subsurface environments.

## Additional file

**Additional file 1: Table S1.** Means of CO_2_ production in PW (produced water) and Sludge enrichments amended with HPAM or PAM as nitrogen (NS) or carbon sources (CS). Pairwise comparisons were conducted by a Tukey–Kramer test after one-way ANOVA analysis. Means with the same letter are NOT significant from each other (Tukey–Kramer test, P>0.05). Controls without polymer are defined as HPAM or PAM free. **Table S2.** Predominant taxa identified at the genus and/or family levels in an oilfield produced water sample (PW) and microbial enrichments derived from the PW that were amended with PAM or HPAM as the sole nitrogen source (only OTUs with relative abundance >0.5% are shown). Unclassified: corresponds to reads that could not be assigned by QIIME at the genus level (the next assigned taxon is specified). OTU = operational taxonomic unit; QIIME = Quantitative Insights Into Microbial Ecology. **Table S3**. Microbial community composition at the genus or family levels showing the number of OTU reads detected for waste water sludge sample (Sludge) and microbial enrichments derived from this sludge sample that were amended with PAM or HPAM as the sole nitrogen source. Unclassified: corresponds to reads that could not be assigned by QIIME at the genus level (the next assigned taxon is specified). OTU = operational taxonomic unit; QIIME = Quantitative Insights Into Microbial Ecology. **Table S4.** Accession numbers and sample names of partial raw sequences that were deposited in GenBank (SUB4856039).
