# Supplementary material for: High temperature utilization of PAM and HPAM by microbial communities enriched from oilfield produced water and activated sludge
Source: AMB Express. 2019 Apr 9;9:46. doi: 10.1186/s13568-019-0766-9 (PMC6456633; doi:10.1186/s13568-019-0766-9)
Supplement: Supplementary file 1 — Additional file 1: Table S1. Means of CO2 production in PW (produced water) and Sludge enrichments amended with HPAM or PAM as nitrogen (NS) or carbon sources (CS). Pairwise comparisons were conducted by a Tukey–Kramer test after one-way ANOVA analysis. Means with the same letter are NOT significant from each other (Tukey–Kramer test, P>0.05). Controls without polymer are defined as HPAM or PAM free. Table S2. Predominant taxa identified at the genus and/or family levels in an oilfield produced water sample (PW) and microbial enrichments derived from the PW that were amended with PAM or HPAM as the sole nitrogen source (only OTUs with relative abundance >0.5% are shown). Unclassified: corresponds to reads that could not be assigned by QIIME at the genus level (the next assigned taxon is specified). OTU = operational taxonomic unit; QIIME = Quantitative Insights Into Microbial Ecology. Table S3. Microbial community composition at the genus or family levels showing the number of OTU reads detected for waste water sludge sample (Sludge) and microbial enrichments derived from this sludge sample that were amended with PAM or HPAM as the sole nitrogen source. Unclassified: corresponds to reads that could not be assigned by QIIME at the genus level (the next assigned taxon is specified). OTU = operational taxonomic unit; QIIME = Quantitative Insights Into Microbial Ecology. Table S4. Accession numbers and sample names of partial raw sequences that were deposited in GenBank (SUB4856039). [file 13568_2019_766_MOESM1_ESM.docx]

**AMB Express**

**Additional Information**

**for**

**High temperature utilization of PAM and HPAM by microbial communities enriched from oilfield produced water and activated sludge**

Carolina Berdugo-Clavijo^1^, Arindom Sen^2^, Mojtaba Seyyedi^2^, Harvey Quintero^3^, Bill O’Neil^3^, and Lisa M. Gieg^1^*

^1^Department of Biological Sciences, University of Calgary and ^2^ Department of Chemical and Petroleum Engineering, Schulich School of Engineering, University of Calgary

2500 University Drive NW, Calgary, AB T2N 1N4, Canada;

^3^Trican Well Service, Calgary, AB, T2P 4G8, Canada

*** Corresponding author:**Lisa M. Gieg
[lmgieg@ucalgary.ca](mailto:lmgieg@ucalgary.ca)

phone: 403-210-7207; FAX 403-289-9311

**Table S1**. Means of CO_2_ production in PW (produced water) and Sludge enrichments amended with HPAM or PAM as nitrogen (NS) or carbon sources (CS). Pairwise comparisons were conducted by a Tukey–Kramer test after one-way ANOVA analysis. Means with the same letter are NOT significant from each other (Tukey–Kramer test, P>0.05). Controls without polymer are defined as HPAM or PAM free.

**Table S2.** Predominant taxa identified at the genus and/or family levels in an oilfield produced water sample (PW) and microbial enrichments derived from the PW that were amended with PAM or HPAM as the sole nitrogen source (only OTUs with relative abundance >0.5% are shown). Unclassified: corresponds to reads that could not be assigned by QIIME at the genus level (the next assigned taxon is specified). OTU = operational taxonomic unit; QIIME = Quantitative Insights Into Microbial Ecology

**Table S3**. Microbial community composition at the genus or family levels showing the number of OTU reads detected for waste water sludge sample (Sludge) and microbial enrichments derived from this sludge sample that were amended with PAM or HPAM as the sole nitrogen source. Unclassified: corresponds to reads that could not be assigned by QIIME at the genus level (the next assigned taxon is specified). OTU = operational taxonomic unit; QIIME = Quantitative Insights Into Microbial Ecology

**Table S4.** Accession numbers and sample names of partial raw sequences that were deposited in GenBank (SUB4856039).
